# Clinical case report: A case of Turner syndrome with Graves’ disease

**DOI:** 10.1097/MD.0000000000019518

**Published:** 2020-03-13

**Authors:** Hongmin Zhang, Xingyu Zhang, Mei Yang

**Affiliations:** Department of Endocrinology, The First People's Hospital of Chongqing Liang Jiang New Area, Chongqing, China.

**Keywords:** Graves’ disease, hypogonadotropic hypogonadism, Turner syndrome

## Abstract

**Introduction:**

The incidence of Hashimoto's thyroiditis among patients who have Turner syndrome (TS) has increased, but Graves’ disease (GD) in patients with TS is rarely reported. Here we report a rare case of TS with GD accompanied by hypogonadotropic hypogonadism.

**Patient concerns:**

We report the case of a 16-year-old girl who complained nervousness, fatigue, marasmus, heat intolerance, sweating, palpitation, and tremor lasting for more than a month. She had no medical history.

**Diagnosis:**

TS was diagnosed of the results of karyotyping demonstrated a gene karyotype of 46, X, i (X)(q10). GD was also diagnosed in this patient following the detection of thyroid function analysis.

**Interventions:**

Methimazole was administered after identification of GD. Due to the absence of secondary sex characteristics, the patient was given a conjugated estrogen preparation for 1 year, followed by the addition of estradiol cyproterone tablets for the onset of menstruation.

**Outcomes:**

The hyperthyroidism symptoms of the patient had improved both clinically and laboratory tests after methimazole therapy. She was treated with estrogen and estradiol cyproterone, and the uterus and secondary sexual characteristics of the patient developed during 1 year follow-up.

**Conclusion:**

TS generally presents as hypergonadotropic hypogonadism. However, hypogonadotropic hypogonadism cannot completely exclude TS. The diagnosis of this disease depends on chromosomal examination. The disease should be detected and treated as early as possible to improve life quality of the patient.

## 1 Introduction

It is well known that Turner syndrome (TS) is among the most common chromosomal abnormalities resulted from structural or numeric abnormalities in the X chromosome.^[1–3]^ It is found in 1/2000 to 1/3000 live-born females.^[4]^ Characteristic physical abnormalities include a short stature, broad chest, webbed neck, kidney abnormalities, cubitus valgus, edema of the hands or feet, cardiac anomalies, gonadal dysgenesis, and delayed puberty.^[1,5]^

Patients with TS suffer from an increasing risk of autoimmune thyroid disorders, celiac disease, vitiligo, psoriasis, type 1 diabetes, adrenocortical insufficiency, juvenile idiopathic arthritis, and inflammatory bowel disease.^[6–9]^ Hashimoto's thyroiditis (HT) is more frequent in patients who have TS.^[10,11]^ From perspectives of pathogenic mechanism in autoimmune thyroiditis, a higher incidence of Graves’ disease (GD) might also be expected in TS patients. However, the connection between GD and this syndrome is significantly more infrequent than expected.^[3,12]^ The aim of our study was to report a case of TS with GD.

## 2 Case presentation

This study was approved by the ethics committee of the First People's Hospital of Chongqing Liang Jiang New Area. All procedures performed in studies involving human participants were in accordance with the ethical standards of the institutional and/or national research committee and with the 1964 Helsinki declaration and its later amendments or comparable ethical standards.

**Figure 1:**
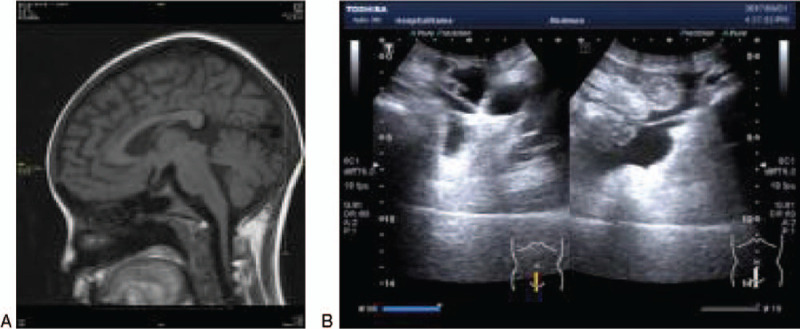
(A) Pituitary MRI revealed partial empty sella. (B) The uterus and both ovaries could not be identified by pelvis ultrasonography. MRI = magnetic resonance imaging.

In 2017, a 16-year-old girl first visited our hospital and complained nervousness, fatigue, marasmus, heat intolerance, sweating, palpitation, and tremor lasting for more than a month. Physical examination revealed smooth, moist, and warm skin; mild exorbitism, and diffused thyroid gland enlargement. Fine finger tremor was not detected. The patient's heart rate was 148 beats/minutes, the blood pressure was 112/66 mm Hg, and precordial systolic murmur of grade 1 was detected. Her height was 132 cm, the weight was 22 kg, and body mass index was 12.6 kg/m^2^. The patient's external genitalia were juvenile, with a pubertal state of A1M1P1 (Tanner staging). Hormonal assays, chromosomal karyotyping, X-ray analysis of bone age, abdominal and pelvic ultrasound, echocardiography, pelvic magnetic resonance imaging (MRI), and pituitary MRI were performed. Her bone age was 12 years old. The results of bone marrow puncture suggested iron deficiency anemia. Pituitary MRI revealed partial empty sella (Fig. 1A). The results of karyotyping demonstrated a gene karyotype of 46, X, i (X)(q10), indicating TS.

Thyroid function analysis presented that the thyroid-stimulating hormone (TSH) level was 0.01 μIU/mL (normal range, 0.38–5.57 μIU/mL), the free (f) T4 level 3.16 ng/dL (normal range, 0.78–1.86 ng/dL), the free T3 level 6.46 pg/mL (normal range, 1.8–3.8 pg/mL), the antithyroglobulin antibody level 2321.7 IU/mL (normal range, 0.00–95.00 IU/mL), the antithyroid peroxidase antibody level 10,000 IU/mL (normal range, 0.00–25.00 IU/mL), and the TSH receptor antibody level >300 IU/L (normal range, 0.00–1.50 IU/L). GD was indicated by the results above, and methimazole was provided. The dose of methimazole was adjusted according to thyroid hormone levels.

**Figure 2:**
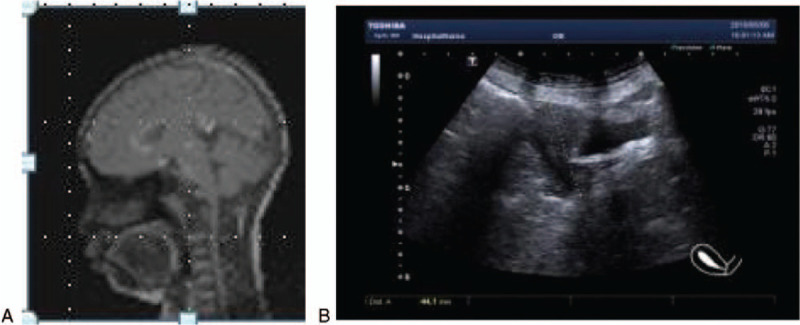
(A) Pituitary-enhanced MRI indicated partially empty sella. (B) A small uterus was detected, and both ovaries could not be identified on pelvis ultrasonograph.MRI = magnetic resonance imaging.

Other hormonal tests demonstrated that the prolactin level was 3.10 ng/mL (reference range, 4.1–28.9 ng/mL) and the testosterone level was 8.95 ng/mL (reference range, 9.81–82.1 ng/mL). The patient's estradiol level was less than 25.00 pg/mL (reference range, 40.7–424.6 pg/mL), the luteinizing hormone level was less than 0.2 mIU/mL, and the level of follicle-stimulating hormone was less than 1.0 mIU/mL. The ovaries and uterus failed to be detected by pelvis ultrasonography (US) (Fig. 1B), MRI, or computed tomography. Due to the absence of secondary sex characteristics, the patient was given a conjugated estrogen preparation for 1 year, followed by the addition of estradiol cyproterone tablets for the onset of menstruation. At the latest follow-up (17 years old), the patient's breasts had developed to Tanner stage 2. The patient's bone age was 13.5, and pituitary-enhanced MRI indicated that she still had partially empty sella (Fig. 2A). Neither ovary could be detected by pelvis US; however, a small uterus was identified (Fig. 2B).

## 3 Discussion

TS has a high incidence of autoimmune diseases, including HT, celiac disease, diabetes mellitus, inflammatory bowel disease, GD, and adrenocortical insufficiency, in girls.^[13,14]^ Excess autoimmune antibodies likely result from X chromosome defects.^[15,16]^ Haploinsufficiency of more than 10 genes located on the X chromosome was related to the immune regulating process, influenced the regulation of the immune response and led to altered immune tolerance.^[15,17]^ Another mechanism suggested the upregulation of pro-inflammatory cytokines contributed to the increasing susceptibility of girls with TS to Autoimmune thyroid disease.^[18]^ The reasons why the thyroid gland was an autoimmune target in TS patients have not yet been clearly identified, but the predilection could be elucidated based on the close connection between the thyroid autoimmunity and female gender.^[19]^

The present patient exhibited a diffused thyroid gland and thyrotoxicosis longer than approximately 1 month. The result of serum analysis was highly positive for antithyroid antibodies.^[12,20]^ In contrast to the well-recognized combination of TS and HT, the combination of TS and GD is rather rare.^[21,22]^ The next issue is why TS accompanied by hyperthyroidism is rare, compared with TS with HT.^[3,23]^ This may be elucidated if typical GD and HT involve different autoimmune processes.^[24,25]^ Since TS belongs to sex chromosome disorder, it is likely that the combination of these 2 disorders is attributed to a specific gene.^[26,27]^ Considering the high susceptibility of patients with TS to autoimmune disease, human leukocyte antigen genes analysis is of vital importance.^[6,28]^ To date, there has been no clear explanation to explain this problem. In conclusion, the connection between GD and TS is far from elucidated, and we cannot exclude that the 2 diseases are just related by chance.^[29]^ In our patient, treatment with thiamazole was continued and the thyroid function has stayed within normal ranges.

TS generally presents as hypergonadotropic hypogonadism,^[30]^ but this patient presented with hypogonadotropic hypogonadism. This may be related to the patient's vacuolar sella turcica, which can compress the pituitary tissue and displace the pituitary stalk, thus leading to a decline in hypophysis function; in addition, this may be related to the patient's malnutrition. The patient was given successively estrogen and estradiol cyproterone treatment to develop the uterus and secondary sexual characteristics.

## 4 Conclusion

Vigilance for TS is required when young girls exhibit hyperthyroidism accompanied by growth retardation and gonadal dysplasia. A decrease in the gonadotropin expression cannot completely exclude TS. The diagnosis of this disease depends on chromosomal examination. The disease should be detected and treated as early as possible to improve the short-term and even long-term life quality of the patient.

## Acknowledgments

The authors are appreciative to AJE (https://www.aje.com/) for language editing.

## Author contributions

**Conceptualization:** Mei Yang.

**Project administration:** Hongmin Zhang, Xingyu Zhang.

**Supervision:** Mei Yang.

**Writing – original draft:** Hongmin Zhang.

**Writing – review & editing:** Hongmin Zhang, Mei Yang.

## References

[1] Stochholm K Juul S Juel K Prevalence, incidence, diagnostic delay, and mortality in Turner syndrome. J Clin Endocrinol Metab 2006;91:3897–902. 16849410 10.1210/jc.2006-0558

[2] Davenport ML Approach to the patient with Turner syndrome. J Clin Endocrinol Metab 2010;95:1487–95. 20375216 10.1210/jc.2009-0926

[3] Livadas S Xekouki P Fouka F Prevalence of thyroid dysfunction in Turner's syndrome: a long-term follow-up study and brief literature review. Thyroid 2005;15:1061–6. 16187915 10.1089/thy.2005.15.1061

[4] Gravholt CH Epidemiological, endocrine and metabolic features in Turner syndrome. Eur J Endocrinol 2004;151:657–87. 15588233 10.1530/eje.0.1510657

[5] Wolff DJ Van Dyke DL Powell CM Working Group of the ACMG Laboratory Quality Assurance Committee. Laboratory guideline for Turner syndrome. Genet Med 2010;12:52–5. 20081420 10.1097/GIM.0b013e3181c684b2

[6] Aversa T Gallizzi R Salzano G Atypical phenotypic aspects of autoimmune thyroid disorders in young patients with Turner syndrome. Ital J Pediatr 2018;44:12. 29343299 10.1186/s13052-018-0447-3 PMC5773039

[7] Aversa T Lombardo F Valenzise M Peculiarities of autoimmune thyroid diseases in children with Turner or Down syndrome: an overview. Ital J Pediatr 2015;41:39. 25971674 10.1186/s13052-015-0146-2 PMC4440559

[8] Morgan T Turner syndrome: diagnosis and management. Am Fam Physician 2007;76:405–10. 17708142

[9] Trovo de Marqui AB Turner syndrome and genetic polymorphism: a systematic review. Rev Paul Pediatr 2015;33:364–71. 25765448 10.1016/j.rpped.2014.11.014 PMC4620965

[10] Wasniewska M Salerno M Corrias A The evolution of thyroid function after presenting with Hashimoto thyroiditis is different between initially euthyroid girls with and those without Turner syndrome. Horm Res Paediatr 2016;86:403–9. 27866202 10.1159/000452722

[11] Bondy CA Turner Syndrome Study Group. Care of girls and women with Turner syndrome: a guideline of the Turner Syndrome Study Group. J Clin Endocrinol Metab 2007;92:10–25. 17047017 10.1210/jc.2006-1374

[12] Wasniewska M Corrias A Messina MF Graves’ disease prevalence in a young population with Turner syndrome. J Endocrinol Invest 2010;33:69–70. 19542755 10.1007/BF03346552

[13] Sybert VP McCauley E Turner's syndrome. N Engl J Med 2004;351:1227–38. 15371580 10.1056/NEJMra030360

[14] Valenzise M Aversa T Corrias A Epidemiology, presentation and long-term evolution of Graves’ disease in children, adolescents and young adults with Turner syndrome. Horm Res Paediatr 2014;81:245–50. 24504143 10.1159/000357130

[15] Alvarez-Nava F Lanes R Epigenetics in Turner syndrome. Clin Epigenetics 2018;10:45. 29636833 10.1186/s13148-018-0477-0 PMC5889574

[16] Gravholt CH Hansen KW Erlandsen M Nocturnal hypertension and impaired sympathovagal tone in Turner syndrome. J Hypertens 2006;24:353–60. 16508584 10.1097/01.hjh.0000200509.17947.0f

[17] Berglund A Cleemann L Oftedal BE 21-hydroxylase autoantibodies are more prevalent in Turner syndrome but without an association to the autoimmune polyendocrine syndrome type I. Clin Exp Immunol 2019;195:364–8. 30372540 10.1111/cei.13231 PMC6378376

[18] Duijnhouwer AL Bons LR Timmers Hv Aortic dilatation and outcome in women with Turner syndrome. Heart 2019;105:693–700. 30368486 10.1136/heartjnl-2018-313716

[19] AbdelMassih AF Attia M Ismail MM Samir M Insulin resistance linked to subtle myocardial dysfunction in normotensive Turner syndrome young patients without structural heart diseases. J Pediatr Endocrinol Metab 2018;31:1355–61. 30433872 10.1515/jpem-2018-0207

[20] Shin JY Kim BH Kim YK Pheochromocytoma as a rare cause of hypertension in a 46 X, i(X)(q10) turner syndrome: a case report and literature review. BMC Endocr Disord 2018;18:27. 29747617 10.1186/s12902-018-0253-3 PMC5946487

[21] Marsudi BA Kartapradja H Paramayuda Cv Loss of DMRT1 gene in a Mos 45,XY,-9[8]/46,XY,r(9)[29]/47,XY,+idic r(9)x 2[1]/46,XY,idic r(9)[1]/46,XY[1] female presenting with short stature. Mol Cytogenet 2018;11:28. 29760778 10.1186/s13039-018-0379-z PMC5941566

[22] Suzuki E Shima H Toki M Complex X-chromosomal rearrangements in two women with ovarian dysfunction: implications of chromothripsis/chromoanasynthesis-dependent and -independent origins of complex genomic alterations. Cytogenet Genome Res 2016;150:86–92. 28099951 10.1159/000455026

[23] Cazzolla AP Lo Muzio L Di Fede O Orthopedic-orthodontic treatment of the patient with Turner's syndrome: review of the literature and case report. Spec Care Dentist 2018;38:239–48. 29846955 10.1111/scd.12295

[24] Gravholt CH Dollerup OL Duval L A rare case of embryonal carcinoma in a patient with Turner syndrome without Y chromosomal material but mutations in KIT, AKT1, and ZNF358 demonstrated using exome sequencing. Sex Dev 2017;11:262–8. 29197878 10.1159/000484398

[25] Hamza RT Raof NA Abdallah KO Prevalence of multiple forms of autoimmunity in Egyptian patients with Turner syndrome: relation to karyotype. J Pediatr Endocrinol Metab 2013;26:545–50. 23446949 10.1515/jpem-2012-0265

[26] Vidmar AP Miyazaki B Sanchez-Lara PA Pitukcheewanont P X-linked hypophosphatemic Rickets, del(2)(q37.1;q37.3) deletion syndrome and mosaic Turner syndrome, mos 45,X/46,X, del(2)(q37.1;q37.3) in a 3-year-old female. J Bone Metab 2017;24:257–61. 29259966 10.11005/jbm.2017.24.4.257 PMC5734952

[27] Goldacre MJ Seminog OO Turner syndrome and autoimmune diseases: record-linkage study. Arch Dis Child 2014;99:71–3. 24064113 10.1136/archdischild-2013-304617

[28] Xue D Cao DH Mu K Mosaic male fetus of Turner syndrome with partial chromosome Y: a case report. J Obstet Gynaecol Res 2018;44:1158–62. 29517175 10.1111/jog.13617

[29] Zhou Q Yao F Wang F A heterozygous mutation in RPGR associated with X-linked retinitis pigmentosa in a patient with Turner syndrome mosaicism (45,X/46,XX). Am J Med Genet A 2018;176:214–8. 29135076 10.1002/ajmg.a.38501

[30] Mohamed SOO Elkhidir IHE Abuzied AIH Prevalence of autoimmune thyroid diseases among the Turner Syndrome patients: meta-analysis of cross sectional studies. BMC Res Notes 2018;11:842. 30486859 10.1186/s13104-018-3950-0 PMC6264051

